# Supplementary material for: Dual RNA-Seq Analysis Reveals Transcriptome Effects during the Salmon–Louse Interaction in Fish Immunized with Three Lice Vaccines
Source: Vaccines (Basel). 2022 Nov 7;10(11):1875. doi: 10.3390/vaccines10111875 (PMC9692469; doi:10.3390/vaccines10111875)
Supplement: Supplementary file 1 [file vaccines-10-01875-s001.zip › Supplementary figures.pdf]

# Supplementary figures

.

A

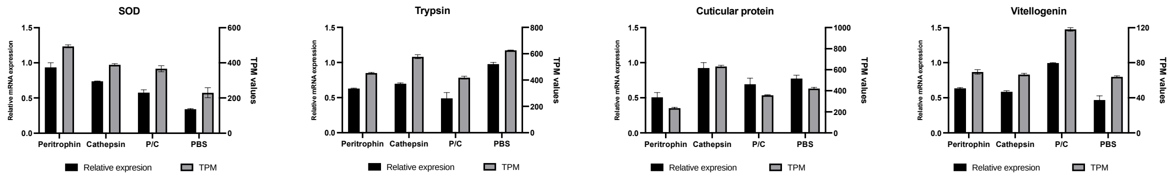


B

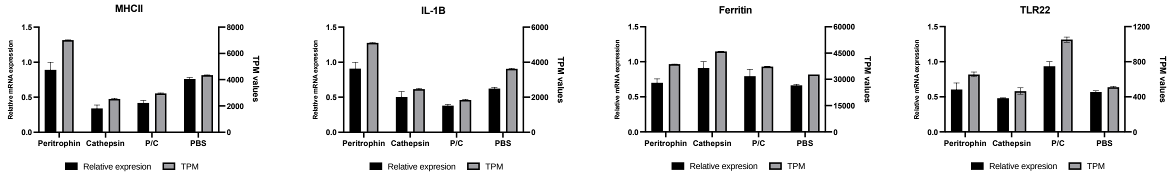


C

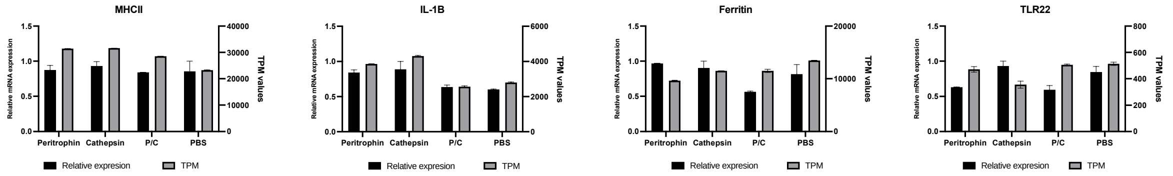


**Figure S1**. RT-qPCR validation of genes expressed in immunized *Salmo salar* and exposed sea lice. A) RT-qPCR validation of genes expressed in sea lice. B) RT-qPCR validation of genes expressed in *S. salar* skin tissue. C) RT-qPCR validation of genes expressed in *S. salar* head kidney tissue.

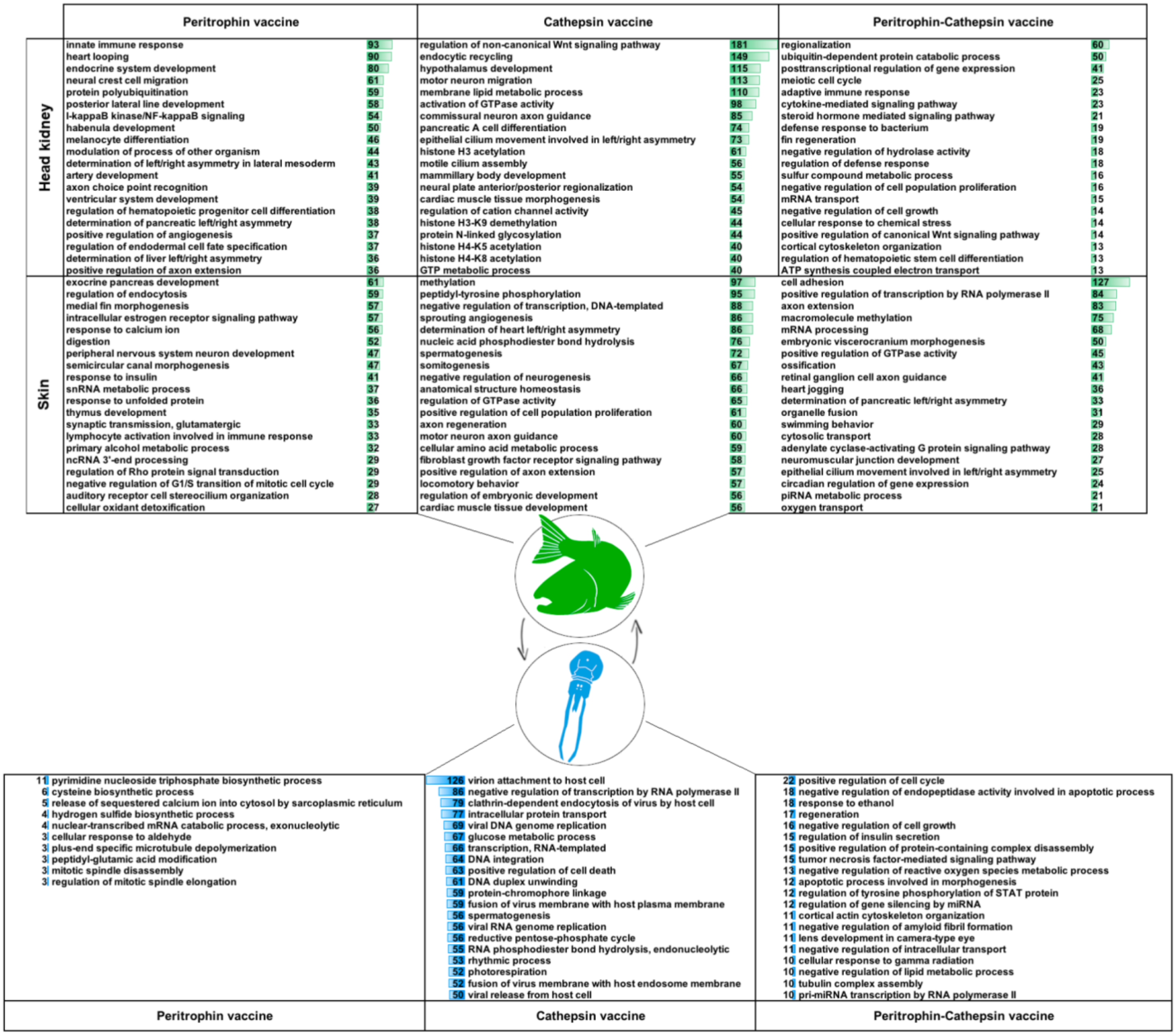


**Figure S2**. GO annotation of Biological Process identifies in genes differentially expressed in vaccinated *S. salar* and *C. rogercresseyi* collected from vaccinated fish. Bars indicate the number of hits for the GO categories.

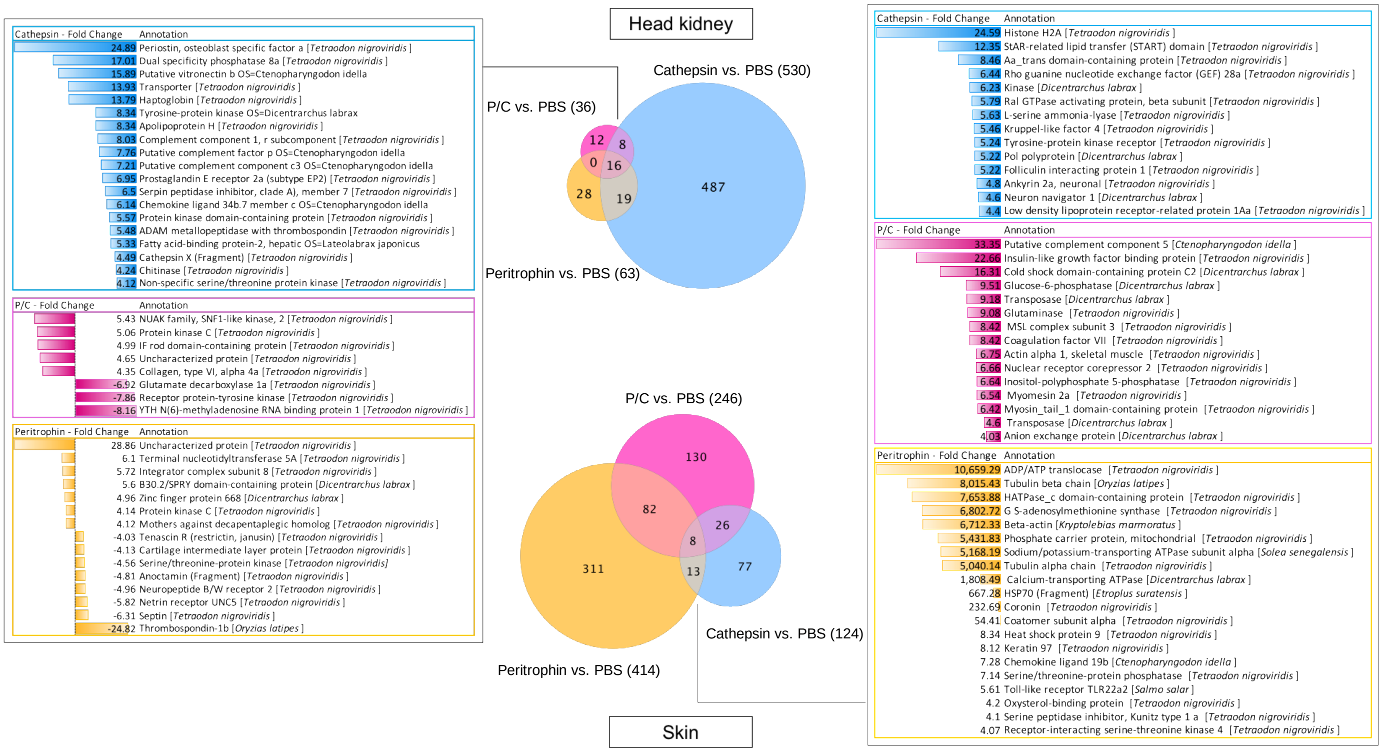


**Figure S3.** Differential expression analysis of genes associated with chromosomal regions with CGE = 100% in vaccinated Atlantic salmons. Venn diagram showing the distribution of contigs differentially expressed. Colored bars indicate the genes fold change: cathepsin in blue, peritrophin in yellow, and purple for the P/C combination.

| | Peritrophin vaccine | | Cathepsin vaccine | | Peritrophin-Cathepsin vaccine | |
|---|---|---|---|---|---|---|
| **Head kidney** | ubiquitin protein ligase activity | 102 | nuclear receptor activity | 162 | ATP binding | 89 |
| | protein kinase binding | 55 | FAD binding | 107 | DNA-binding transcription factor activity | 73 |
| | iron-sulfur cluster binding | 49 | methylated histone binding | 80 | sequence-specific DNA binding | 71 |
| | growth factor activity | 42 | hemi-methylated DNA-binding | 77 | calcium ion binding | 52 |
| | ATP-dependent chromatin remodeler activity | 38 | 5'-3' exonuclease activity | 76 | protein homodimerization activity | 39 |
| | NADH dehydrogenase (ubiquinone) activity | 35 | acetylglucosaminyltransferase activity | 65 | sodium ion transmembrane transporter activity | 25 |
| | ribosome binding | 28 | damaged DNA binding | 55 | guanyl-nucleotide exchange factor activity | 22 |
| | toxin activity | 27 | oxidoreductase activity | 52 | actin filament binding | 18 |
| | oxidoreductase activity | 27 | histone H3-methyl-lysine-9 demethylase activity | 44 | calmodulin binding | 18 |
| | fibroblast growth factor binding | 26 | histone H4-methyl-lysine-20 demethylase activity | 40 | electron transfer activity | 18 |
| | amide binding | 26 | enzyme activator activity | 36 | cytoskeletal motor activity | 16 |
| | solute:sodium symporter activity | 25 | histone H3-tri/di-methyl-lysine-27 demethylase activity | 32 | oxidoreductase activity, acting on NAD(P)H | 16 |
| | glutathione oxidoreductase activity | 24 | NADPH:sulfur oxidoreductase activity | 28 | ATP-dependent chromatin remodeler activity | 14 |
| | mRNA 3'-UTR binding | 24 | palmitoyl-(protein) hydrolase activity | 19 | isomerase activity | 14 |
| | fibroblast growth factor-activated receptor activity | 23 | histone glutaryltransferase activity | 19 | transition metal ion transmembrane transporter activity | 13 |
| | DNA primase activity | 23 | histone succinyltransferase activity | 19 | cell adhesion molecule binding | 13 |
| | vascular endothelial growth factor-activated receptor activity | 23 | arginine transmembrane transporter activity | 14 | RNA helicase activity | 12 |
| | cysteine-type endopeptidase activity | 22 | acetylcholinesterase activity | 13 | fibroblast growth factor binding | 10 |
| | L-aspartate transmembrane transporter activity | 22 | | | estrogen receptor activity | 10 |
| | platelet-derived growth factor alpha-receptor activity | 20 | | | ATPase-coupled cation transmembrane transporter activity | 9 |
| **Skin** | structural constituent of ribosome | 103 | ATP binding | 181 | ATP binding | 239 |
| | proton transmembrane transporter activity | 61 | sequence-specific DNA binding | 131 | GTPase activator activity | 89 |
| | rRNA binding | 37 | DNA-binding transcription factor activity | 129 | calmodulin binding | 56 |
| | copper ion binding | 35 | calcium ion binding | 114 | histone binding | 53 |
| | antioxidant activity | 27 | zinc ion binding | 108 | heme binding | 51 |
| | NAD+ binding | 18 | protein serine kinase activity | 95 | S-adenosylmethionine-dependent methyltransferase activity | 50 |
| | protein-lysine 6-oxidase activity | 17 | protein homodimerization activity | 82 | modification-dependent protein binding | 37 |
| | protein kinase C binding | 16 | actin binding | 78 | N-methyltransferase activity | 33 |
| | cysteine-type endopeptidase inhibitor activity | 15 | lyase activity | 65 | extracellular matrix structural constituent | 25 |
| | ATPase-coupled intramembrane lipid transporter activity | 15 | microtubule binding | 64 | thiol-dependent deubiquitinase | 24 |
| | laminin binding | 14 | endonuclease activity | 59 | ubiquitin binding | 23 |
| | triglyceride lipase activity | 14 | heparin binding | 57 | beta-catenin binding | 22 |
| | quinone binding | 12 | iron-sulfur cluster binding | 56 | oxygen binding | 21 |
| | retinoic acid-responsive element binding | 9 | guanyl-nucleotide exchange factor activity | 56 | oxygen carrier activity | 21 |
| | long-chain-3-hydroxyacyl-CoA dehydrogenase activity | 8 | photoreceptor activity | 55 | RNA helicase activity | 20 |
| | dodecenoyl-CoA delta-isomerase activity | 8 | protein tyrosine phosphatase activity | 54 | phosphatidylinositol phosphate binding | 19 |
| | MAP-kinase scaffold activity | 7 | exonuclease activity | 52 | 2-oxoglutarate-dependent dioxygenase activity | 19 |
| | U3 snoRNA binding | 7 | single-stranded DNA binding | 52 | histone demethylase activity | 18 |
| | | | growth factor activity | 51 | microfilament motor activity | 17 |
| | | | fibroblast growth factor-activated receptor activity | 49 | G protein-coupled amine receptor activity | 17 |

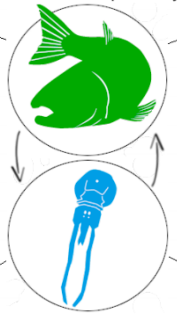

| | | | | | |
|---|---|---|---|---|---|
| 6 | tryptophan synthase activity | 288 | ATP binding | 22 | tubulin binding |
| 6 | fatty acid binding | 168 | zinc ion binding | 18 | actin filament binding |
| 5 | carbon-nitrogen ligase activity, with glutamine as amido-N-donor | 138 | magnesium ion binding | 13 | SH3 domain binding |
| 4 | chemokine (C-C motif) ligand 5 binding | 134 | heme binding | 13 | cytoskeletal motor activity |
| 4 | acetyl-CoA C-acyltransferase activity | 124 | structural constituent of ribosome | 11 | structural constituent of eye lens |
| 3 | phosphatidylcholine floppase activity | 108 | GTP binding | 11 | amyloid-beta binding |
| 3 | tubulin-dependent ATPase activity | 101 | calcium ion binding | 9 | L-ascorbic acid binding |
| 3 | troponin T binding | 101 | RNA polymerase II sequence-specific DNA binding | 8 | peptidylamidoglycolate lyase activity |
| | | 93 | serine-type endopeptidase activity | 8 | peptidylglycine monooxygenase activity |
| | | 92 | cytochrome-c oxidase activity | 6 | 3 iron, 4 sulfur cluster binding |
| | | 80 | protein homodimerization activity | 6 | maltose binding |
| | | 75 | 4 iron, 4 sulfur cluster binding | 5 | STAT family protein binding |
| | | 71 | GTPase activity | 5 | succinate dehydrogenase (ubiquinone) activity |
| | | 69 | protein serine/threonine kinase activity | 4 | iron-sulfur cluster carrier activity |
| | | 68 | NADH dehydrogenase (ubiquinone) activity | 4 | voltage-gated potassium channel activity |
| | | 66 | RNA-directed 5'-3' RNA polymerase activity | 4 | steroid 11-beta-monooxygenase activity |
| | | 64 | DNA-directed DNA polymerase activity | 4 | corticosterone 18-monooxygenase activity |
| | | 58 | DNA helicase activity | 3 | mitogen-activated protein kinase p38 binding |
| | | 57 | RNA helicase activity | 3 | calcium-independent phospholipase A2 activity |
| | | 57 | NADP binding | 3 | palmitoyl-CoA hydrolase activity |
| | **Peritrophin vaccine** | | **Cathepsin vaccine** | | **Peritrophin-Cathepsin vaccine** |

**Figure S4.** GO annotation of Molecular Function identifies in genes differentially expressed in vaccinated *S. salar* and *C. rogercresseyi* collected from vaccinated fish. Bars indicate the number of hits for the GO categories.

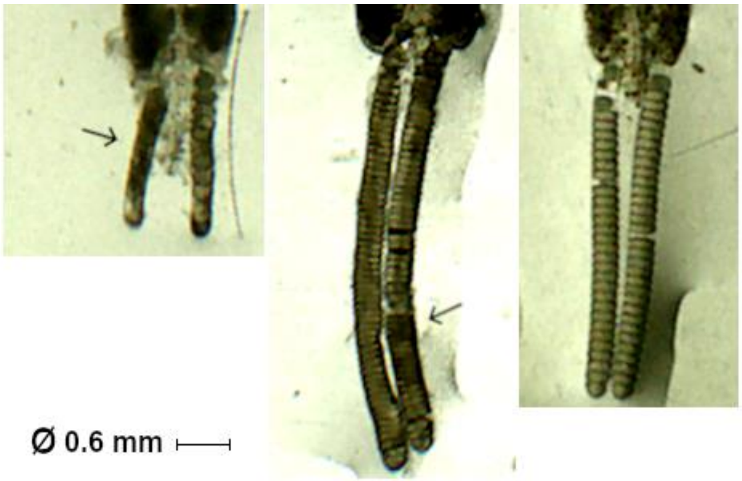


**Figure S5.** Phenotypic malformations of female *Caligus rogercresseyi* exposed to (A) cathepsin, (B) peritrophin, and (C) PBS-vaccinated fish. Images were taken under the same conditions with a 2X objective lens. The arrows indicate alterations concerning the control groups. The scale of 0.6 mm indicated by the black circle is equivalent in all the images of the figure
