# Supplementary material for: Dual RNA-Seq Analysis Reveals Transcriptome Effects during the Salmon–Louse Interaction in Fish Immunized with Three Lice Vaccines
Source: Vaccines (Basel). 2022 Nov 7;10(11):1875. doi: 10.3390/vaccines10111875 (PMC9692469; doi:10.3390/vaccines10111875)
Supplement: Supplementary file 1 [file vaccines-10-01875-s001.zip › Supplementary TableS1.pdf]

Table S1. Primers list used for RT-qPCR analysis.

Organism	**Primer Name**	**Gene**	**Sequence 5'-3'**	**Tm**	**Efficiency (%)**	**Reference**
Salmo salar	EFa_F	*Elongation factor- (reference gene)*	TGCTGGTGGTGTGGGTGAGT	60	95.18	[19]
	EFa_R		CCTCAAACCGCTTCTGGCTGT			
	IL1B_F6	*Interleukin-1B*	GATCTGGAGGTATCCCATCA	60	122,36	[19]
	IL1B_R6		CACAGCACTCTCCAGCAAGA			
	MHCII_F	*Major histocompatibility complex II*	CTCCTCAAAGGACCTGCAGG	60	104,72	[19]
	MHCII_R		TCAGGACCTTTGTTCCAGGC			
	TLR22_3_F	*Toll-like recePtor 22*	TGCCTTCTAACCCTCTCCCT	61	91,36	[19]
	TLR22_3_R		CGCTGCTCTCAGACAGGAAG			
	FerritinM_F1	*Ferritin M*	TATCACCACGATTGCGAAGC	60	102,66	[19]
	FerritinM_R1		CTCGTCGCTGTTCTCCTTGA			
Caligus rogercresseyi	Cr_β-tubulin_2F	*β - tubulin (reference gene)*	TTTGTTGTGTGAGCTCTGGG	60	92,8	[15]
	Cr_β-tubulin_2R		GCTGATCTCCGAAAACTTGC			
	qCr_SOD_F	*Superoxide dimutase*	TCCACATGCAACACGCGCTCCAG	60	109.29	[27]
	qCr_ SOD _R		GCGTGCCCTGGTCGTACATGCCG			
	CRtryp-58-F1	*Trypsin*	CAACTTCAGACCAAATGAC	60	99.8	[27]
	CRtryp-58-R1		CATAGAGTTCGTGGTAGAT			
	conting56379_F1	*Cuticle protein*	AATCCAAGTCCATCAGCGAC	58	100	[16]
	conting56379_F1		CTCCAACATCAACTCTGCCA			
	Cr_Vit_F1	*Vitellogenin*	GTCTGCCACGTTGAGAAGGA	58	113	This study
	Cr_Vit_R1		CGAGGAGTCCGAATCTGACG			
